# 

**Published:** 1916-11

**Authors:** 


					﻿Vol. XXXII
NOVEMBER, 1916
No. 5
TEXAS
Medical JWrnal
ESTABLISHED JtJLY. 1885
PUBLISHED MONTHLY AT AUSTIN, TEXAS, BY
MRS. F. E. DANIEL, - K - Publisher and Managing Editor
Subscription, 81.00 a Year, In Advance.	- Single Copies, 10 Cents
Entered atthe Postofflce at Austin, Texas, as second class mail matter.
ft
<
a
I
I
				

